# Development of New Collagen/Clay Composite Biomaterials

**DOI:** 10.3390/ijms23010401

**Published:** 2021-12-30

**Authors:** Maria Minodora Marin, Raluca Ianchis, Rebeca Leu Alexa, Ioana Catalina Gifu, Madalina Georgiana Albu Kaya, Diana Iulia Savu, Roxana Cristina Popescu, Elvira Alexandrescu, Claudia Mihaela Ninciuleanu, Silviu Preda, Madalina Ignat, Roxana Constantinescu, Horia Iovu

**Affiliations:** 1 Advanced Polymer Materials Group, Politehnica University of Bucharest, 1-7 Polizu Street, 011061 Bucharest, Romania; rebeca.leu@upb.ro; 2 Collagen Department, Leather and Footwear Research Institute, 93 Ion Minulescu Street, 031215 Bucharest, Romania; albu_mada@yahoo.com (M.G.A.K.); madalina.fleancu@yahoo.com (M.I.); rodica.roxana@yahoo.com (R.C.); 3 National Research & Development Institute for Chemistry and Petrochemistry, ICECHIM, Spl. Independentei Nr. 202, 6th District, 060021 Bucharest, Romania; catalina.gifu@icechim-pd.ro (I.C.G.); elviraalexandrescu@yahoo.com (E.A.); claudia.ninciuleanu@icechim-pd.ro (C.M.N.); 4 Department of Life and Environmental Physics, Horia Hulubei National Institute of Physics and Nuclear Engineering, 077125 Magurele, Romania; savu_diana@yahoo.com (D.I.S.); Roxana.popescu@nipne.ro (R.C.P.); 5 Institute of Physical Chemistry “Ilie Murgulescu”, Romanian Academy, Spl. Independentei 202, 6th District, 060021 Bucharest, Romania; predas01@yahoo.co.uk; 6 Chemical Sciences Section, Academy of Romanian Scientists, 54 Splaiul Independentei, 50085 Bucharest, Romania

**Keywords:** clay, type II collagen, biomaterials

## Abstract

The fabrication of collagen-based biomaterials for skin regeneration offers various challenges for tissue engineers. The purpose of this study was to obtain a novel series of composite biomaterials based on collagen and several types of clays. In order to investigate the influence of clay type on drug release behavior, the obtained collagen-based composite materials were further loaded with gentamicin. Physiochemical and biological analyses were performed to analyze the obtained nanocomposite materials after nanoclay embedding. Infrared spectra confirmed the inclusion of clay in the collagen polymeric matrix without any denaturation of triple helical conformation. All the composite samples revealed a slight change in the 2-theta values pointing toward a homogenous distribution of clay layers inside the collagen matrix with the obtaining of mainly intercalated collagen-clay structures, according X-ray diffraction analyses. The porosity of collagen/clay composite biomaterials varied depending on clay nanoparticles sort. Thermo-mechanical analyses indicated enhanced thermal and mechanical features for collagen composites as compared with neat type II collagen matrix. Biodegradation findings were supported by swelling studies, which indicated a more crosslinked structure due additional H bonding brought on by nanoclays. The biology tests demonstrated the influence of clay type on cellular viability but also on the antimicrobial behavior of composite scaffolds. All nanocomposite samples presented a delayed gentamicin release when compared with the collagen-gentamicin sample. The obtained results highlighted the importance of clay type selection as this affects the performances of the collagen-based composites as promising biomaterials for future applications in the biomedical field.

## 1. Introduction

The design of collagen biomaterials for use as implants poses various challenges for tissue engineers [1]. Biomaterials offer great advantages for medical therapies, facilitating a good response for clinical problems [2,3]. Biopolymers represent an important resource for biomedical applications because of their characteristics, for example, in cell adhesion, proliferation and compatibility with diverse categories of drugs, and of different chemical structure [4,5,6]. The forms of mixed biopolymers commonly used are hydrogels, membranes, spheres, fibers, and sponges.

Hydrogels are 3D crosslinked polymers structures with high capacity to absorb and retain important water amounts without the degradation of their tridimensional network. Hydrogels can be synthesized from natural, synthetic, or combined polymer chains. Hydrogels can serve as matrices for the obtaining of composite biomaterials, targeting adequate structures and morphologies beneficial for biomedical application [7,8,9,10]. Because of their availability and versatility, natural polymers such as collagen, alginate, chitosan, hyaluronic acid, and cellulose are mostly preferred for their use in various formulations and mixtures. These are regularly used for a series of remedies in regenerative medicine and drug delivery systems [11,12].

Collagen represents the foremost component of extracellular matrix with a unique triple helical structure, and it is renowned for its low antigenicity, abundance in vertebrate organism, exceptional biocompatibility, and desired biodegradability, unique features that recommend it as an exceptional biomaterial for medical application [13,14]. All these properties promote collagen for use in various biomaterials as wound dressings, bone substitutes, antithrombogenic surfaces, and ophthalmologic collagen shields. Moreover, collagen found application in tissue engineering, for skin replacement and artificial blood vessels and valves [15]. Type II collagen can be found in the extracellular matrix of the articular cartilage as the most important structural element alongside proteoglycans and others specific collagens [16]. Moreover, type II collagen is produced in vitreous, embryonic cornea, and retinal nerve membrane [17]. It is extensively used in food industry, cosmetics, and biomedical and pharmaceuticals applications. Nowadays, the requirement for type II collagen in biomaterials field is progressively growing [18,19]. However, collagen-based biomaterials applications are frequently limited by inadequate thermal stability and mechanical strength. Nevertheless, these drawbacks can be mitigated through the use of cross-linking agents, interpenetration with synthetic polymers, or through the addition of inorganic fillers [20,21].

Currently, several clays are being utilized in medicine and pharmaceuticals as active components or excipients, as well as in cosmetics for creams and emulsion preparation [22]. The majority of clays is obtained from alkaline volcanic sediments using a hydrothermal process. Clays are composed of very fine particles with mixed metal ions. Mostly, they include phyllosilicates such as hydrous silicates of aluminum (Al), zinc (Zn), magnesium (Mg), iron (Fe), and smaller amounts of other metal ions [23]. The structure of clays can be viewed in a platelet form having less than 2 μm in diameter and less than 10 nm in thickness. Furthermore, every level includes minimum one silica (SiO_2_) tetrahedron (T) succeeded by one alumina (Al_2_O_3_) octahedron (O). Clays are divided into three large groups: serpentine-kaolin composed by the halloysite species with chemical formula Al_2_Si_2_O_5_(OH)_4_·2H_2_O; smectites that comprise two categories: montmorillonite with chemical formula Si_12_Mg_8_O_30_(OH)_4_(OH_2_)_4_·8H_2_O and laponite (synthetic hectorite) with chemical formula Na^+0.7^[(Si_8_Mg_5.5_Li_0.3_)O_20_(OH)_4_]^−0.7^; and Sepiolite-polygorskite that is composed of sepiolite with chemical formula Si_12_Mg_8_O_30_(OH)_4_(OH_2_)_4_·8H_2_O, having an architecture like montmorillonite with small differences in its morphology [24].

In the last years, polymer/clay nanocomposites incorporating drugs have been studied to provide new strategies for various applications in the regenerative medicine field [25]. The most studied nanoclays used in the synthesis of collagen-based biomaterials envisaged for medical area are laponite, halloysite, and montmorillonite [25,26,27]. For example, Yu et al. [28] prepared a chitosan-collagen/organomontmorillonite composite loaded with *Callicarpa nudiflora* as a wound dressing membrane. Moreover, Wang et al. [29] obtained composite fibers doped with amoxicillin using poly (lactic-co-glycolic acid) copolymer and laponite. Another set of polymer/clay nanocomposites based on multilayered polylactic acid/halloysite/gentamicin membranes for bone regeneration, was obtain by Pierchala and collaborators [30]. In a previous study [31], a polyester and acrylate-based composite with hydroxyapatite and halloysite nanotubes was developed and characterized envisaging medical applications. Another hybrid collagen-based hydrogel with embedded montmorillonite (Dellite HPS, Dellite 67G, Cloisite 93A) nanoparticles was developed by Nistor et al. [32].

Montmorillonite (MMT) is a natural biomaterial that has grown relevant due to its high availability, good internal surface area, high cation exchange capability, good adsorption and swelling ratio, excellent biocompatibility. Additionally, montmorillonite is an FDA approved material [33,34,35]. Several research studies proved that the introduction of clays into polymers matrices not only influenced the mechanical properties of the new designed composites, but also the modulated swelling ratio, degradation rate, and drug release [19,36,37]. The literature data about the conformational change in type II collagen structure induced by Cloisites, which is essential for understanding the new properties of clay mineral–collagen nanocomposites, are deficient. 

**Scheme 1:**
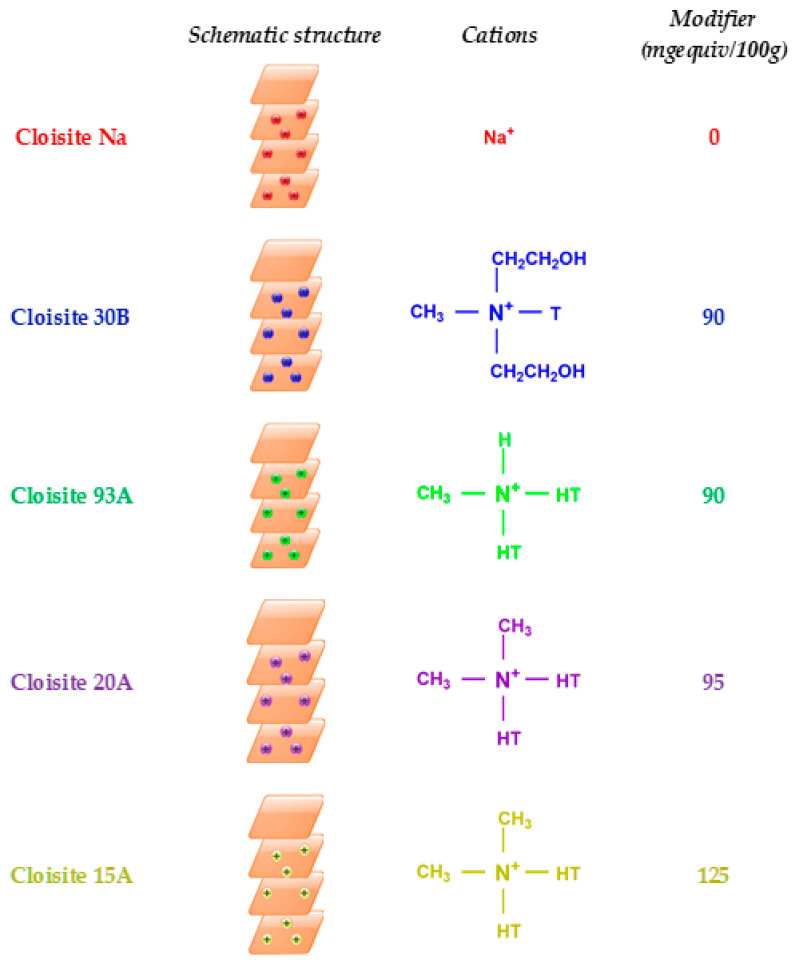
Schematic structures of the different types of mineral clays.

In this regard, the purpose of the present study was to obtain, characterize, and investigate novel composite biomaterials for semi-hard tissue based on type II collagen with several types of clays that will be further loaded with gentamicin bioactive agent. The structures of the different types of Cloisites are provided in the scheme below (Scheme 1). The commercial clays: Cloisite 20A, Cloisite 15A, Cloisite 93A, and Cloisite 30B are organically modified montmorillonites with quaternary ammonium salts of fatty acids, while Cloisite Na represents the commercial name of montmorillonite without any organomodification [38]. Through organomodification process or cation exchange reaction, the hydrophilic surface of montmorillonite is converted to hydrophobic state. Thus, hydrophobic functional moieties beneficially increase the interactions between clay and nonpolar molecules [39]. 

Although several studies regarding the use of different natural or synthetic clays with collagen are available, none of them provides a systematic comparison between the uses of Cloisite clay type series (Cloisite Na, Cloisite 20A, Cloisite 15A, Cloisite 93A, and Cloisite 30B) in the synthesis and characterization of type II collagen-based biomaterials. Therefore, our study offers valuable information about the importance of clay type selection in the preparation of collagen-based biomaterials when a fixed concentration of clay is used. We expect that the inclusion of mineral clays to provide enhanced mechanical stability serving as reinforcing agents for the biopolymer matrix. Moreover, our study includes investigations about the influence of clay type on the release of gentamicin from gentamicin loaded collagen–clay composites and bactericidal and biocompatibility studies of the prepared nanocomposite materials. To the best of our knowledge, our study is the first systematic report investigating the inclusion of natural or functionalized clay in a type II collagen matrix for cartilaginous tissue regeneration, representing a first for the composite community.

## 2. Results and Discussion

### 2.1. FTIR Analyses

**Figure 1:**
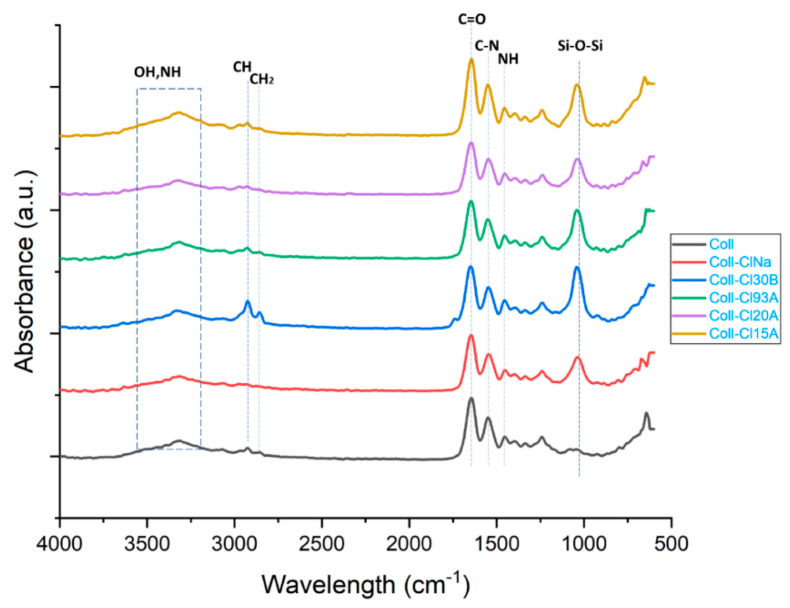
FTIR spectra of the obtained collagen/clay composite biomaterials.

The structure of the composite biomaterials was determined by infrared spectrometry and are presented in Figure 1. The spectra of collagen/clay composite biomaterials indicated the characteristic infrared bands of specific components. 

The collagen macromolecule presents a specific triple helix conformation characterized, in infrared spectra, by the amide bands [21]. The peaks around 3319 cm^−1^ and 2928 cm^−1^, are attributed to amide A and B bands, mostly associated with the NH stretching vibrations, OH groups, and CH asymmetric vibration [20,32]. The amide I band at 1648 cm^−1^ is assigned to the stretching vibrations of peptide C=O groups. The amide II is attributed with the peak at 1551 cm^−1^ arises to CN stretching vibrations. The Amide III band positioned at 1240 cm^−1^ is assigned to the NH bending vibrations from amide linkages [32]. 

FTIR spectra confirmed the inclusion of clay in the collagen polymeric matrix without any denaturation of triple helical conformation where the specific peaks of clay corresponding to Si-O-Si stretching vibration and CH_2_ groups from the hydrocarbonate chains of the clay organic modifiers were found in the collagen/clay composite biomaterials at 1040–1048 cm^−1^ and, respectively, at 2800–2900 cm^−1^.

### 2.2. X-ray Diffraction Analyses

**Figure 2:**
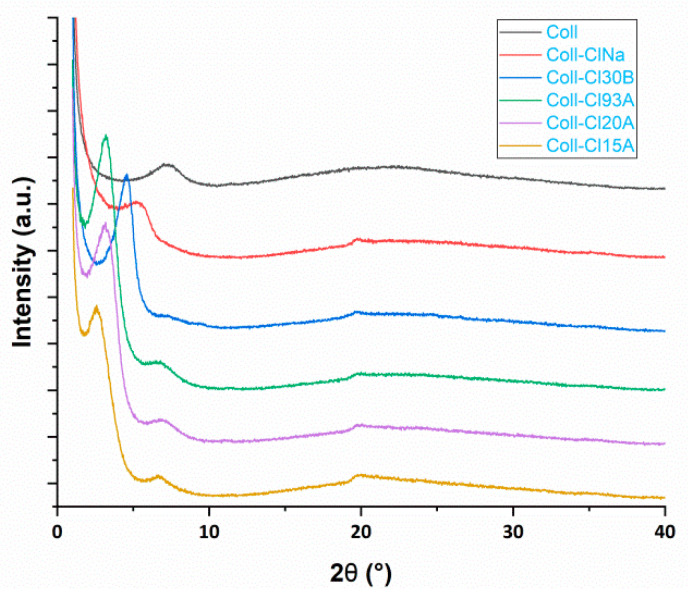
X-ray diffraction patterns of composite samples.

The X-ray diffraction patterns of collagen/clay composite biomaterials are presented in Figure 2. Collagen diffraction patterns exhibit two major diffraction lines at two Bragg angles as follows: at ~7.5° a diffraction line generally assigned to triple helix molecular chains and at ~20° a diffraction line attributed to unordered components of collagen [40].

Usually, the mineral clay nanoparticles have a great miscibility with polymeric network and they can basically allow collagen insertion between clay layers [33]. According to XRD analyses, all the composite samples revealed a slight change in the 2-theta values pointing toward a homogenous distribution of clay layers inside the collagen matrix with the obtaining of mainly intercalated collagen–clay structures. Cloisite sodium sample exhibited a better compatibility with the collagen matrix as revealed by the broadening and shifting of Cloisite sodium specific peak. 

### 2.3. SEM Analyses

**Figure 3:**
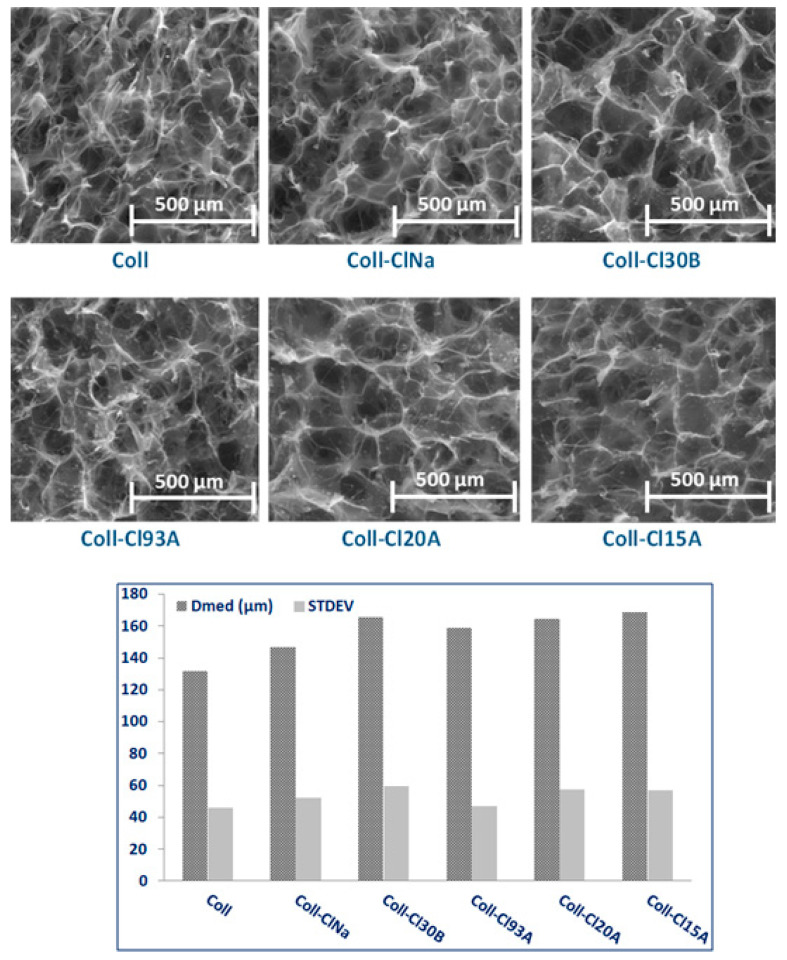
SEM images of collagen/clay composite biomaterials (250×) and calculated average pore diameter with standard deviation.

SEM images (Figure 3) indicated a porous structure with interconnected pores for all the analyzed collagen-based samples.

The neat collagen sample presented a spongious structure with smaller pores than collagen/clay composite materials. The addition of mineral clay nanoparticles in polymeric matrix produces a disturbed porous structure, and somewhat larger pores with clay aggregates were visualized. 

In order to quantify the possible changes in pore sizes, the average pore diameter (Dmed) was calculated using Scandium software after measuring 100 pores of each collagen-based sample. The presence of the clays in the architectural structure of collagen affected the polymeric network assembly according the calculated Dmed. The porosity of collagen/clay composite biomaterials varied depending on clay nanoparticles sort. Thus, Coll sample presented the minimum pore size with Dmed = 132 µm while the samples with mineral clays exhibited slightly higher values starting from Dmed = 147 µm for unmodified clay sample (Coll-ClNa) to Dmed ~160 to 170 µm for organomodified mineral clays containing samples [33]. These findings are in good agreement with other studies that observed greater pore sizes when clay particles are added to the biopolymer matrix [41,42,43].

### 2.4. Thermogravimetrical Analyses

**Figure 4:**
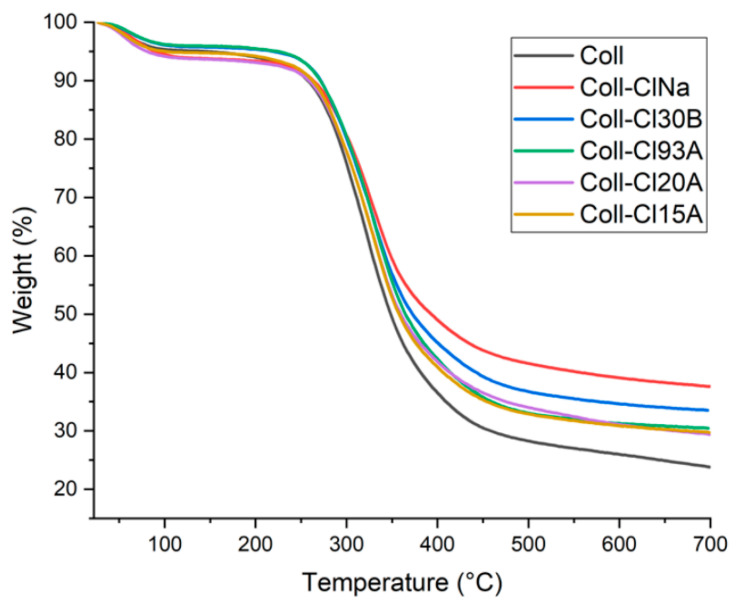
TGA curves of collagen/clay composite biomaterials.

In order to confirm that the addition of mineral clay nanoparticles in polymeric matrix improved the thermal stability of collagen biomaterials, TGA investigations were performed. Thermal analyses could provide useful information firstly regarding mineral clay dispersion inside collagen matrix and secondly, insights related to supplementary physical and/or chemical crosslinking within the networks [44,45]. The resulted TGA thermograms of the collagen/clay composite biomaterials are presented in Figure 4.

The thermal stability of obtained composites has a significant role in defining the quality of the medical devices [28]. According to the obtained thermograms, for all the collagen/clay composite samples, the presence of the clays promoted an increase in their thermal stability. These results may indirectly point toward a denser structure possibly related to physical interactions established between nanoclays and polymer molecules [46]. TGA thermograms showed a multistep degradation for all samples. The first thermal transitional step appears in the range 45–150 °C and can be associated to the loss of water from the polymeric material [20,32]. The second thermal stage was recorded in the range 150–350 °C, which is associated with a gradual decomposition stage, correspondingly to the irreversible denaturation process [20]. 

In Table 1 are summarized the properties of the collagen/clay composite biomaterials.

Moreover, the increasing residual mass of composite biomaterials, according to the data presented in Table 1, confirms the embedding of mineral clays into the polymeric matrix [32]. The differences in the residual mass of composite materials are related with the organomodifier’s presence, which gives greater residual mass for samples containing less hydrophobic clays. These findings are similar to those reported by Leon-Mancilla et al. [47] and the same enhanced thermal effect was demonstrated by other studies on polymer/clay nanocomposites. This phenomenon is generally related to the impediment of transport of gases in the nanocomposite sample caused by clay nanoplatelets that can act as a barrier retarding the decomposition process [48]. Moreover, these results could indirectly point interaction between clay nanoparticles and collagen matrix.

### 2.5. Swelling Studies

**Figure 5:**
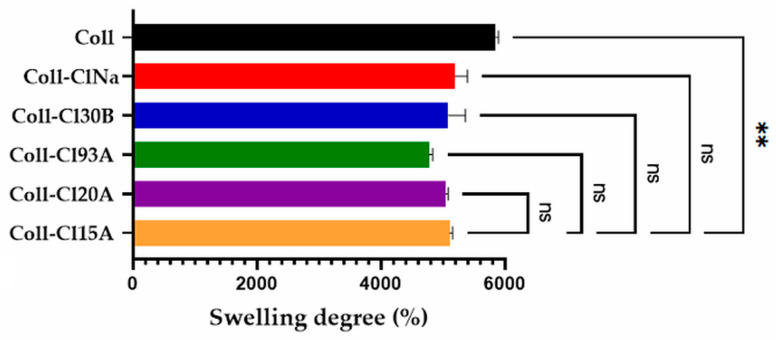
Equilibrium swelling degree of collagen-based samples (ns *p* > 0.05, ** *p* ≤ 0.01).

Generally, the biomaterials used for tissue regeneration must have specific characteristics for the development and maintenance of an optimal environment and water loss from the damaged tissue at an optimal ratio that is influenced by the water absorption capacity [28]. Swelling studies of all collagen samples indicated a very high ability to retain water, swelling degree ranging from 4500 to 5900% with respect to xerogel samples (Figure 5). 

The addition of mineral clay nanoparticles in the collagen polymeric matrix decreased the PBS equilibrium swelling degree of nanocomposite sample. This fact could be a consequence of the replacement of collagen with clay (in the synthesis stage) that did not absorb as much water as related collagen networks [49]. The swelling ability could be restricted also due the additional crosslinking caused by hydrogen bonding. Several researchers demonstrated clay–polymer interactions between the charged surface of clay nanosheets and certain functional groups from the polymer structure, yielding stronger networks [1]. 

Among nanocomposites samples, a very interesting is the fact that the Cl93A samples displayed the lowest swelling degree. Analyzing the structure of the several clays, Cloisite 93A structure presents a distinct sulfate counter anion of quaternary ammonium salt when compared with the other clay types, which present chloride as counter anion. Therefore, it is very likely that Cl93A could have generated a more crosslinked nanocomposite structure because of sulfate binding, which involves direct hydrogen bonding with positively charged amino groups from collagen polymeric networks, as other studied demonstrated [50].

Overall, the inclusion of clay into the collagen networks led to the limitation of hydrogel swelling most likely due collagen–clay interactions. 

### 2.6. Biodegradation

For biomedical applications an ideal scaffold should present a suitable degradation rate to match the regenerating process of the damaged tissue [49]. The biodegradation of collagen structure can be achieved using collagenase solutions, which are composed of enzymes that are able to destroy the collagen triple helix conformation under the biological conditions of pH and temperature [51].

**Figure 6:**
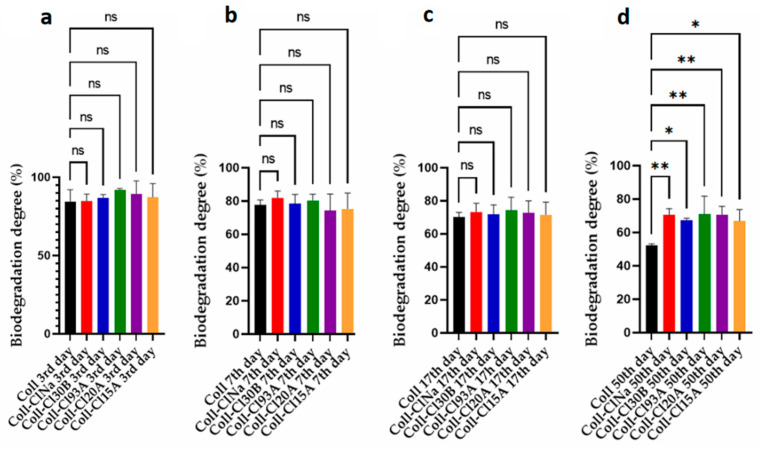
Biodegradation degree as function of time for the composite samples: (**a**). third day; (**b**). 7th day, (**c**). 17th day; (**d**). 50th day (ns *p* > 0.05, * *p* ≤ 0.05, ** *p* ≤ 0.01).

All the hydrogel samples presented a good stability for a long period of time, retaining more than 50% of the samples’ mass after a long period of 50 days (Figure 6).

The addition of the mineral clays led to a decrease in the grade of enzymatic degradation by conserving more of the matrix ultrastructure comparing to the neat collagen sample. The interaction between mineral clays and collagen ultrastructure may consume some hydrophilic groups such as NH_2_, which prevented macromolecular hydrolyzation, reinforcing the stability of the obtained biomaterials [49]. The most stable hydrogels were Coll-Cl93A and Coll-Cl20A nanocomposite samples. Biodegradation findings are also supported by swelling studies, which indicated a more crosslinked structure due additional H bonding brought on by nanoclays, especially Cl93A clay type.

The obtained nanocomposites could be suitable scaffolds for cartilaginous tissue regeneration as these retain their mass up to more than 65% after 50 days. Therefore, we expect that the biodegradable scaffolds will remain stable up to 3 months, the minimum period considered for cartilage regeneration [52]. 

### 2.7. Mechanical Tests

**Figure 7:**
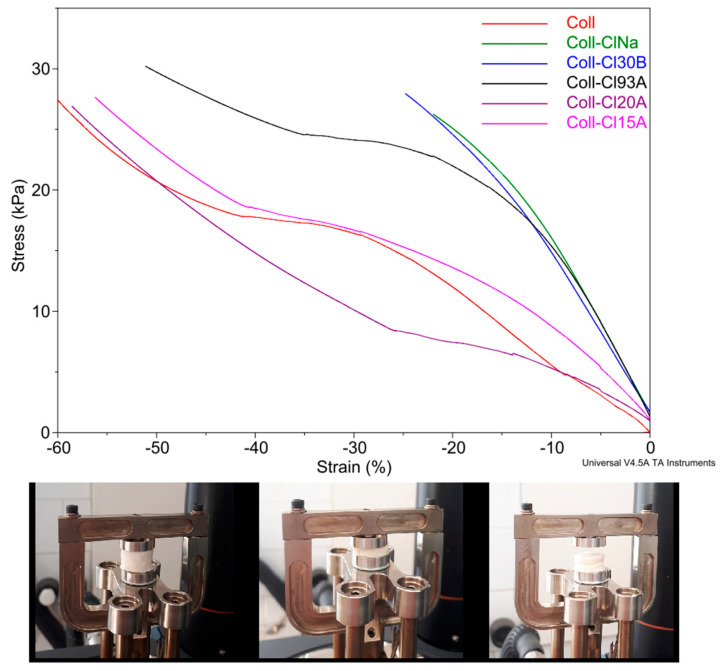
DMA mechanical properties of dried collagen/clay composite biomaterials.

Collagen/clay composite samples gave rise to different mechanical behaviors when compared to the neat collagen sample. Earlier studies also demonstrated that layered silicates induce enhanced mechanical properties when included in different polymer matrices. However, these properties are strongly dependent on clay concentration and platelet distribution into the polymeric matrix [53]. In our case, the addition of mineral clay in the polymer matrix improved the mechanical properties of the dried samples (Figure 7). Increased mechanical stability and a stronger structure were evidenced, and the nanoclay platelets acted as an elastic solid under stress conditions. 

Amongst the five clays used in the synthesis, ClNa produced the most resistant nanocomposite sample to compression. The Coll-Cl30B dried sample had a similar behavior as Coll-ClNa, while the composite obtained with the most hydrophobic clays had a lower resistance under the stress applied compared to the pure collagen sample. This behavior was probably due to the presence of different hydrophobic modifiers, which induced preferential distribution of clay platelets inside the collagen matrix, causing mostly intercalated clay structures as observed by X-ray diffractograms [54]. 

Moreover, the collagen dried sample had the highest distortion under stress, while the composites samples, especially those obtained with hydrophilic clays, deformed much less (almost three times) than the neat sample (Table 2). Very probably, the organic groups induced a slight elasticity of the composite samples, which recovered better and faster after mechanical stress (Table 2).

**Figure 8:**
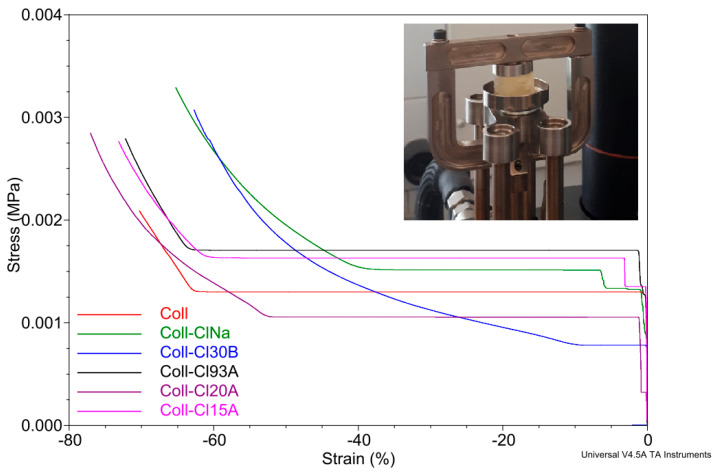
DMA stress–strain curves for the collagen/clay composite hydrogels swollen in PBS.

The compression tests performed on wet samples revealed that the presence of the inorganic filler also led to significant improvements in the mechanical strength of the nanocomposite samples (Figure 8). Especially, the presence of ClNa and Cl30B clays led to a significant increase in compressive strength of the composite wet samples against collagen neat sample. Thus, the same behavior from xerogel samples was also preserved to the hydrogel samples, the composite samples being more resistant to mechanical stress than the pure collagen, with emphasis on the samples obtained with the most hydrophilic clays.

These results are in good agreement with earlier studies where layered silicates included in a polymer matrix tend to increase the intermolecular forces and dissipate the energy in the whole material under stress conditions [49,53]. Moreover, a higher dispersion of clay nanoplatelets and their interaction with the polymeric matrix were demonstrated to have direct consequences on the compressive strength of the final hydrogel-based nanocomposite materials [54]. 

Considering that the compression modules of human articular cartilage may vary from 0.1 to 2 MPa, depending on location, DMA results indicate that the synthesized hydrogel nanocomposites scaffolds could withstand the mechanical environment of the cartilaginous tissue [55].

### 2.8. Drug Release

**Figure 9:**
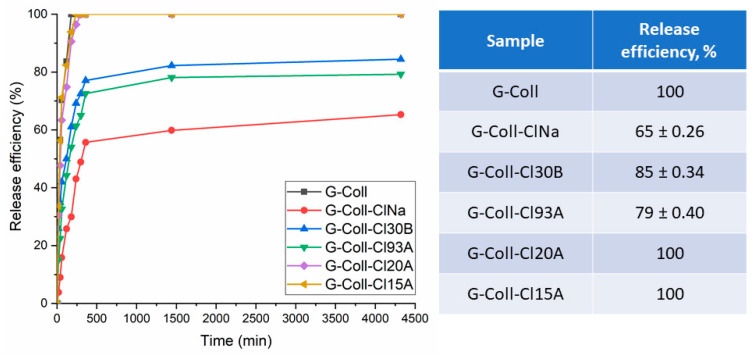
Gentamicin release profile as a function of time.

Gentamicin is one of the most commonly utilized and tested antibiotics in drug delivery systems, and furthermore it has previously been accepted for clinical use [56]. Gentamicin is an antibiotic obtained from *Micromonispora purpurea,* which prevents infection, being efficient against a broad spectrum of Gram-positive and Gram-negative bacteria species [57]. Moreover, gentamicin was demonstrated to promote faster healing process when used along halloysite clay [58]. Considering these aspects, gentamicin was selected and used in our study as a model drug to evaluate clay influence over drug delivery process. The in vitro gentamicin kinetic profiles from collagen/clay composite biomaterials were represented as a drug cumulative released percentage as a function of time (Figure 9).

The release profiles had the same trend for all the samples loaded with gentamicin. Thus, the samples presented an initial burst release in the first hours, followed by a gradually and prolonged drug delivery over the next 72 h. It is worth mentioning that all nanocomposite samples presented a delayed gentamicin release when compared with the collagen-gentamicin sample. Thus, the most rapid release was recorded for the samples G0-Coll followed by G-Coll-Cl20A and G-Coll-Cl15A. Further, the samples obtained with the most hydrophilic clays, G-Coll-Cl30B, G-Coll-Cl93A, and G-Coll-ClNa presented the most retarded burst effect. The cumulative gentamicin released percentage after 72 h has varied between 60 and 80% depending on the type of clay. This extended drug release offers a local shielding antibacterial effect over a longer period of time, essential for tissue repair, and is correlated with the degradation results.

### 2.9. Antimicrobial Activity

**Figure 10:**
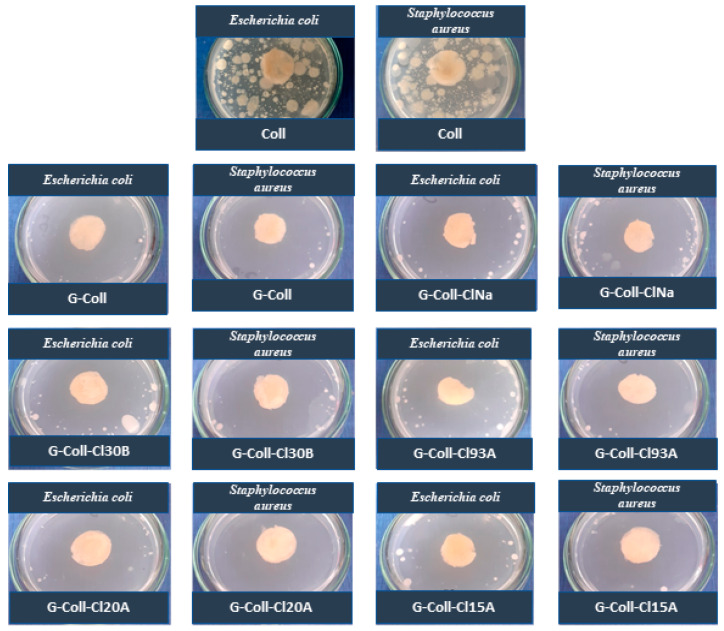
Antimicrobial activity against *Escherichia coli* and *Staphylococcus aureus.*

The most significant feature of biomaterials is the anticipation of microorganisms’ infection as bacteria [28]. The collagen/clay composite biomaterials loaded with gentamicin were tested for microbial activity against two bacterial strains, including *Escherichia coli* and *Staphylococcus aureus* according to SR EN ISO 20645/2005—Control of the antibacterial activity. The evaluation of the samples is based on the absence or presence of bacterial multiplication in the contact area between the inoculum and the sample and on the appearance of a possible inhibition zone around the samples (Figure 10) [59].

An insufficient effect was obtained for the sample without gentamicin and inclusion of mineral clays (Coll) which did not present antimicrobial activity against the bacterial strains. The inhibitions areas produced by all the formulations loaded with gentamicin showed diameters ranging between 6 and 10.5 mm when tested against *Staphylococcus aureus* and 3.5 and 11 mm against *Escherichia coli* after 24 h of incubation. Additionally, it can be observed that the largest zone of inhibition for *Staphylococcus aureus* was presented by the sample G-Coll-Cl20A (10.5 mm), and the largest zone of inhibition for *Escherichia coli* was exhibited by the sample G-Coll (11 mm), which does not have any addition of mineral clay nanoparticles in polymeric matrix. These results are in good agreement with gentamicin release studies where for G-Coll, G-Coll-Cl15A, and G-Coll-Cl20A samples, the most intense burst release and greater amount of gentamicin was recorded within 24 h, thus allowing an effective inhibition of the bacterial strains.

The results summarized in Table 3 revealed that all composite samples loaded with gentamicin presented microbiological activity and do not allow the development of aerobic germs for any of the bacteria tested.

### 2.10. Cellular Viability—MTT Tests

**Figure 11:**
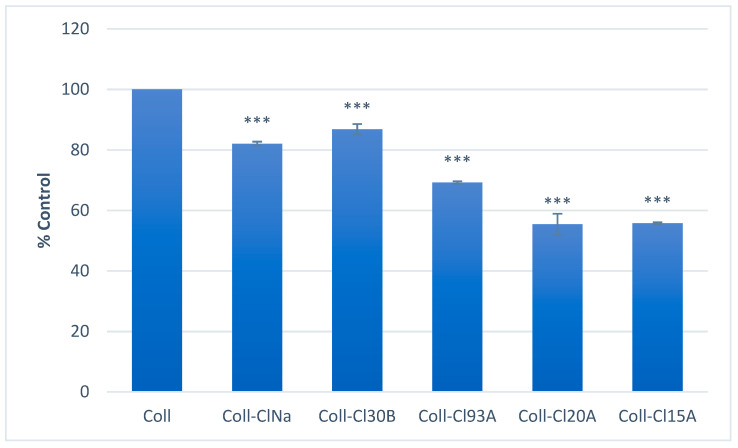
Biological evaluation of the obtained samples, *** p ≤ 0.001.

In order to test the biocompatibility of the new collagen composites we used the MTT assay, which is recognized as a proper method for measuring the cytotoxicity of new biomaterials [60]. The results depicted in the Figure 11 revealed a viability of 82.1 ± 0.61%; 86.8 ± 1.75; 69.3 ± 0.39; 55.4 ± 3.51; and 55.76 ± 0.36 for Coll-ClNa; Coll-Cl30B; Coll-Cl93A; Coll-Cl20A, and Coll-Cl15A, respectively. Thus, the results depicted in the Figure 11 revealed that the Coll-ClNa, Coll-CI30B, and Coll-CI93A composites presented an accepted viability (higher than 70%) in comparison with the control Coll composite. These composites are non-toxic so they could be recommended for biomedical application (ISO 10993-12:2009(E) standard (Biological evaluation of the medical devices. Part 5).

In order to test the biocompatibility of the new collagen composites, we used the MTT assay, which is recognized as a proper method for measuring the cytotoxicity of new biomaterials [60]. 

The results depicted in the Figure 11 revealed that the Coll-ClNa, Coll-CI30B, and Coll-CI93A composites presented an accepted viability (higher than 70%) in comparison with the control Coll composite. These composites are non-toxic so they could be recommended for biomedical application (ISO 10993-12:2009(E) standard (Biological evaluation of the medical devices. Part 5). Our results could be related with organic modifier type, which, in a certain concentration, could influence the safety profile of the resulted collagen-based nanocomposite materials. Thus, the biological evaluation of the composites was found in good agreement with literature data, which suggested that a good biocompatibility occurs only in composites samples with a specific concentration of clay in the polymeric matrices [44]. The specific concentration refers to the recipe used in the synthesis and could range from 0.01 to 7% *w/v* [43].

## 3. Materials and Methods

### 3.1. Materials

Type II collagen gel was obtained from bovine cartilage by alkaline treatments. Each batch of cartilage was supplied from the same cattle farm and type II collagen gels with the same characteristics were extracted by following a patented protocol in the Collagen Department, Skin, and Footwear Research Institute [61]. Gentamicin was purchased from Fluka (Milwaukee, WI, USA). Type I collagenase extracted from *Clostridium histolyticum* was procured from Sigma-Aldrich, Darmstadt, Germany, and glutaraldehyde (GA) from Merck, Darmstadt, Germany. The nanoclays were received from Southern Clay Products (Gonzales, TX, USA,).

### 3.2. Preparation of New Collagen/Clay Composite Biomaterials

Mineral clays were solubilized in ultrapure water and were kept under magnetically stirring for 15 h. Afterwards, the clay dispersion was ultrasonicated for 2 min. Type II collagen and the gentamicin solution were added and homogenized, at 24 °C, to obtain 100 g of gel for each sample in accordance with the composition given in Table 4.

**Figure 12:**
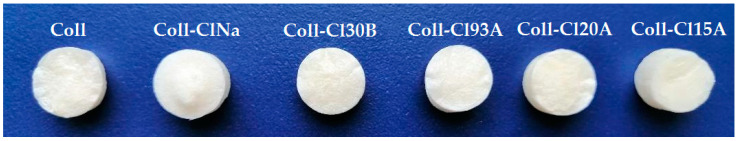
Obtained collagen/clay composite biomaterials (without gentamicin).

The composite hydrogels were freeze-dried using Delta 2–24 LSC (Martin Christ, Osterode, Germany) instrument and spongious forms were obtained and characterized. The obtained 3D composite biomaterials can be observed in Figure 12.

The freeze-dried composite scaffolds were evaluated by Fourier-transform infrared spectroscopy, X-ray diffraction, scanning electron microscopy, differential scanning calorimetry, thermo gravimetric analysis, swelling ratio, biodegradation ratio, mechanical tests, drug release, antimicrobial tests, and cellular viability.

### 3.3. Methods

#### 3.3.1. Fourier-Transform Infrared Spectroscopy (FTIR)

FTIR analysis was accomplished on a Vertex 70 Bruker FTIR spectrometer (Billerica, MA, USA) using an attenuated total reflectance (ATR) addition. For all the obtained biomaterials, the FTIR spectra were recorded in the ATR-FTIR method (in triplicate) at a resolution of 4 cm^−1^ in the 600–4000 cm^−1^ wavenumber range.

#### 3.3.2. X-ray Diffraction (XRD)

The obtained biomaterials were analyzed using an X-ray diffractometer (RigakuUltima IV, Tokyo, Japan) with CuKα radiation (λ = 1.5406 Å), operated at 40 kV and 30 mA. The assessment was performed in uninterrupted mode, at ~23 °C ± 1 and atmospheric pressure. The data were collected over the 2thetawith 1–50° and a scanning speed of 1°/min. The samples were evaluated in a dried powder form. 

#### 3.3.3. Scanning Electron Microscopy (SEM)

All the samples were studied using an environmental scanning electron microscopy (ESEM-FEI Quanta 200, Eindhoven, The Netherlands). Moreover, to obtain the secondary electrons images, a gaseous secondary electron detector (GSED) was utilized with the established parameters: 25–30 kV accelerating voltage, magnifications between 2000 and 5000× for the section of obtained biomaterials and the pressure on 2 torr (vacuum conditions).

#### 3.3.4. Thermo Gravimetric Analysis (TGA)

The thermal properties of obtained collagen/clay composite biomaterials were evaluated in triplicate with a NETZSCH TG 209 F1 Libra instrument (Selb, Germany) (controlled atmosphere with a flow rate of nitrogen about 20 mL/min, scanning from 25 to 700 °C and a heating rate of 10 °C/min). Instead of analysis, all composite biomaterials were assessed, the mass ranging between 4 and 5 mg and then they were introduced into aluminum containers. 

#### 3.3.5. Swelling Ratio

The swelling ratio of the obtained composites was evaluated by incubation in ultrapure water and temperature of 37 °C. After a predefined time, the samples were taken out and surface adsorbed water was removed by filter paper. The swelling ratio was definite as the ratio of weight increase (w—w0) to the initial weight (w0). Each sample was tested in triplicate.

#### 3.3.6. Biodegradation

The biodegradation behavior of each the obtained samples was assessed by placing the specimens (already swelled in PBS) in a collagenase solution. The hydrogels were maintained at 37 °C for up to 50 days. The wet samples were weighted periodically in order to determine the biodegradation degree. The measurements were performed in triplicate. The biodegradation degree of the hydrogels was calculated as the ratio of weight decrease (w—w0) reported to the initial weight (w0).

#### 3.3.7. Mechanical Tests

Dynamic mechanical analysis of the samples was achieved using a DMA Q800 (TA Instruments, New Castle, DE, USA). Measurements were made at 37 °C, in compression mode, using round sponge samples with a diameter of 15 mm and a thickness of 10 mm. All the equilibrium swelled samples were compressed with a ramp force of 0.01 N/min, from 0.01 to 1.5 N. The dried samples were compressed with a Ramp force of 0.1 N/min from 0.01 to 5 N. The method used to evaluate both dried samples and hydrogel samples was the Compression modulus. All the tests were realized in triplicate.

#### 3.3.8. Drug Release

In vitro drug release kinetic of gentamicin from the design scaffolds was investigated by immersion in containers with a volume of 15 mL ultrapure water. The recipients were additionally introduced in an orbital mixer (Benchmark Scientific, Sayreville, NJ, USA) at 300 rpm, and 37 °C. A 5 mL sample was collected at fixed time intervals and then examined by UV–VIS spectroscopy (SHIMADZU UV-3600 instrument). To preserve a constant volume, after each sampling, 5 mL of fresh ultrapure water were added to every flask. The release efficiency (*RE*) was determined using Equation (1) [62]:

(1) $$RE\left(\%\right)=\frac{amount\;of\;released\;GE}{amount\;of\;loaded\;GE}\times 100$$

#### 3.3.9. Antimicrobial Activity

The control of the antimicrobial activity was tested against the *Staphylococcus aureus* (Gram positive) and *Escherichia. coli* (Gram negative) strains. All the steps of this evaluation were described in our previous work [63]. The samples, placed on the surface of the nutrient medium were analyzed after 24 h of incubation at 37 °C. Bioactivity was determined by determining the diameter of inhibition zones in (mm). Each measurement was repeated for three times and the mean of the diameter of the inhibition areas was calculated.

#### 3.3.10. Cellular Viability—MTT Tests

In order to assess the biological effect of the collagen samples, the MG63 cell line (CLS) was used. The cells were cultured in Earle’s minimum essential medium (MEM) containing L-glutamine (Biochrom, Merck Milipore, Burlington, MA, USA) and supplemented with 10% fetal bovine serum (FBS), 1% penicillin and streptomycin antibiotics, and 1% non-essential amino acids in standard conditions of temperature and humidity (37 ± 2 °C, 5 ± 1% CO_2_ and more than 90% humidity). 

The samples were sterilized by gamma irradiation. Afterwards, 2.5 x 10^5^ cells in 500 µL were directly seeded onto each sample. The cells were allowed to attach for about 30 min and afterwards cell culture medium was added in order to cover the whole structure. The cells were incubated in standard conditions of temperature and humidity during 5 days.

The cellular viability was quantitatively measured using the MTT tetrazolium-salt assay (Serva, Heidelberg, Baden-Wuerttemberg, Germany). For this, at the corresponding time-point, the medium was removed and gently replaced with fresh culture medium containing 10% MTT solution (5 mg/mL in PBS). The cells were incubated for another 3 h in standard conditions, and afterwards the supernatant was replaced with DMSO in order to solubilize the grown formazan crystals. The absorbance corresponding to each sample was measured at 570 nm wavelength using a microplate reader. 

In order to eliminate any possible interferences, two “Blanks” samples with and without the material and no cells, were used (according to ISO 10993-12 and ISO 10993-5). The absorbance of the blank containing only the solubilizing agent (DMSO) and the absorbance of the blank containing the material and DMSO were mostly the same, results showing that the material does not interfere with the absorbance measurement.

All experiments were performed in triplicate and the data was presented as mean ± SEM. The statistical analysis was performed using a two-tailed Student’s test, where values of * *p* ≤ 0.05, ** *p* ≤ 0.01, *** *p* ≤ 0.001 were considered as statistically significant.

## 4. Conclusions

Novel Collagen/Clay nanocomposite biomaterials were prepared using five different types of clay. Morphological analyses demonstrated spongiest structures with increased pore dimensions as a result of nanoclay embedding. Mainly intercalated collagen–clay structures with a homogenous distribution of clay layers inside the collagen matrix were obtained as demonstrated by XRD analyses. FTIR spectra confirmed the inclusion of clay in the collagen polymeric matrix without any denaturation of triple helical conformation. The addition of clay nanoparticles into the collagen matrix promoted improved mechanical properties and decreased biodegradation and swelling ratios when compared with the neat collagen sample. The in vitro gentamicin kinetic profiles revealed retarded burst release of gentamicin as function of clay type. All the samples presented good microbiological activity after the inclusion of mineral clay nanoparticles not allowing the development of aerobic germs for any of the bacteria tested. Cellular viability tests showed good biocompatibility of the novel Coll-ClNa, Coll-CI30B, and Coll-CI93A collagen/clay composites.

Thus, our paper presents a preliminary study that aimed to investigate several types of clay of fixed concentration. Following the valuable results obtained, ClNa, Cl30B, and Cl93A will be further used for the development of reinforced hydrogels by varying their concentrations where their influence will be investigated in terms of antimicrobial and drug release properties. 

Based on the results presented in our study, the performances of the new collagen-based composites recommend them as promising biomaterials for future applications in the biomedical field.

## Figures and Tables

**Table 1:** Thermogravimetric properties of the collagen/clay composite biomaterials.

Sample	T_10%_ (°C)	T_50%_ (°C)	Water Loss Step	Thermal Degradation Step	Residual Mass (%)
			T_max%_ (°C)	T_max%_ (°C)	
Coll	257.6	348.5	56.3	319.3	23.82
Coll-ClNa	263.7	393.2	64.8	327.2	37.59
Coll-Cl30B	271.6	372.7	61.0	326.1	33.51
Coll-Cl93A	271.2	364.2	64.3	329.0	30.44
Coll-Cl20A	259.0	359.7	56.3	326.6	29.41
Coll-Cl15A	262.7	357.6	58.7	326.5	29.76

**Table 2:** Behavior of dried samples under stress applied. Revert to the initial form after stress condition.

Sample	Sample Distortion under Stress Condition (Force Applied = 5 N), (%)	Revert after 10 s, (%)	Revert after 30 min, (%)
Coll	62.79	56.45	88.02
Coll-ClNa	20.99	90.66	99.12
Coll-Cl30B	25.68	89.95	94.34
Coll-Cl93A	49.80	68.15	90.52
Coll-Cl20A	58.63	85.12	93.80
Coll-Cl15A	56.08	56.45	88.02

**Table 3:** Evaluation of the antimicrobial activity of the composite samples loaded with gentamicin.

Sample	Inhibition Area (mm)	Bacterial Strain	Evaluation	SD (Standard Deviation) for Three Determinations
Coll	Absent	*Staphylococcus aureus*	Insufficient effect	0
	Absent	*Escherichia coli*	Insufficient effect	0
G-Coll	10	*Staphylococcus aureus*	Satisfactory effect	0.3
	11	*Escherichia coli*	Satisfactory effect	0.1
G-Coll-ClNa	7.5	*Staphylococcus aureus*	Satisfactory effect	0.1
	8.5	*Escherichia coli*	Satisfactory effect	0.1
G-Coll-Cl30B	6	*Staphylococcus aureus*	Satisfactory effect	0.1
	5	*Escherichia coli*	Satisfactory effect	0.1
G-Coll-Cl93A	9.5	*Staphylococcus aureus*	Satisfactory effect	0.1
	3.5	*Escherichia coli*	Satisfactory effect	0.1
G-Coll-Cl20A	10.5	*Staphylococcus aureus*	Satisfactory effect	0.1
	9	*Escherichia coli*	Satisfactory effect	0.2
G-Coll-Cl15A	10	*Staphylococcus aureus*	Satisfactory effect	0.1
	6	*Escherichia coli*	Satisfactory effect	0.1

**Table 4:** The composition of the new collagen/clay composite biomaterials.

Sample	Collagen (%)	ClNa (%)	Cl30B (%)	Cl93A (%)	Cl20A (%)	Cl15A (%)	Gentamicin (GE) (%)	GA (%)
Coll	1.5	-	-	-	-	-	-	0.16
Coll-ClNa	1.5	0.375	-	-	-	-	-	0.16
Coll-Cl30B	1.5	-	0.375	-	-	-	-	0.16
Coll-Cl93A	1.5	-	-	0.375			-	0.16
Coll-Cl20A	1.5	-	-	-	0.375		-	0.16
Coll-Cl15A	1.5	-	-	-	-	0.375	-	0.16
G-Coll	1.5	-	-	-	-	-	0.2	0.16
G-Coll-ClNa	1.5	0.375	-	-	-	-	0.2	0.16
G-Coll-Cl30B	1.5	-	0.375	-	-	-	0.2	0.16
G-Coll-Cl93A	1.5	-	-	0.375	-	-	0.2	0.16
G-Coll-Cl20A	1.5	-	-	-	0.375	-	0.2	0.16
G-Coll-Cl15A	1.5	-	-	-	-	0.375	0.2	0.16

All the percentages are reported to 100 g sample.

## Data Availability

Not applicable.
